# Fault Diagnosis Method for Main Pump Motor Shielding Sleeve Based on Attention Mechanism and Multi-Source Data Fusion

**DOI:** 10.3390/s25061775

**Published:** 2025-03-13

**Authors:** Nengqing Liu, Xuewei Xiang, Hui Li, Zhi Chen, Peng Jiang

**Affiliations:** 1 State Key Laboratory of Power Transmission and Transformation Equipment Technology, Chongqing University, Chongqing 400044, China; 202211021049@stu.cqu.edu.cn (N.L.); cqulihui@cqu.edu.cn (H.L.); jiangpeng@stu.cqu.edu.cn (P.J.); 2 National Key Laboratory of Nuclear Reactor Technology, Nuclear Power Institute of China, Chengdu 610213, China; chenzhinpic@126.com

**Keywords:** shielding sleeve failure, main pump motor, fault diagnosis, attention mechanism, multi-source data fusion, multi-scale features

## Abstract

The operating environment of the shielding sleeve of the main pump motor is complex and changeable, and it is affected by various stresses; so, it is prone to bulging, cracking, and wear failure. The space where it is located is narrow, making it difficult to install additional sensors for condition monitoring. The existing methods have difficulty in taking into account the advantages of multiple aspects, such as the in-depth extraction of multi-scale data features, multi-source data fusion, and attention mechanisms, thus failing to achieve fault diagnosis for the failure of the shielding sleeve. Therefore, this paper proposes a fault diagnosis method for the shielding sleeve based on the attention mechanism and multi-source data fusion. The proposed method is suitable for scenarios where the fault characteristics of single data sources are not obvious and multi-scale and multi-source data need to be fused collaboratively. This method takes the measurable data (torque, rotational speed, voltage, and current) of the main pump motor operation as input signals. First, a multi-scale convolutional neural network based on the attention mechanism (AM-MSCNN) is established to extract rich multi-scale features of the data, and the spatial and channel attention mechanisms are used to fuse the multi-scale features. Then, on the basis of the AM-MSCNN, a convolutional neural network structure based on the attention mechanism for multi-scale and multi-source data fusion (AM-MSMDF-CNN) is proposed to further fuse the primary fusion features of different channels of torque, rotational speed, voltage, and current. Finally, the BP algorithm and the cross-entropy loss function are used to conduct fault diagnosis and classification on the fused features to complete the fault diagnosis of the shielding sleeve failure. To verify the effectiveness of the proposed method, experimental verification was carried out using datasets generated by finite element simulation and a small-scale equivalent prototype. By comparing it to methods such as the one-dimensional convolutional neural network (1D-CNN), Bagging Ensemble Learning, Random Forest, and Support Vector Machine (SVM), it was found that for the simulation data and experimental data, the accuracy of the AM-MSMDF-CNN is 5–10% and 10–15% higher than that of the other methods, demonstrating the superiority of the method proposed in this paper.

## 1. Introduction

The main pump motor is one of the key equipment in pressurized water reactor nuclear power plants and the only rotating mechanical equipment in the reactor coolant system [1]. As an important component of the main pump motor, the failure of the shielding sleeve may lead to a series of serious performance problems and safety hazards. According to statistics, the number of failures of the shielding sleeve accounts for about 35% of the total failures of the main pump. The bulging failure of the shielding sleeve may cause mechanical friction between the rotor and the stator, resulting in mechanical wear and damage of the motor components. The rupture or wear of the shielding sleeve may lead to coolant leakage, reducing the cooling efficiency of the motor. As a result, the heat of the reactor core cannot be discharged, causing major accidents such as core meltdown and radioactive leakage, which pose a serious threat to the safety of equipment and personnel. Early detection of the shielding sleeve failure can avoid the occurrence of major accidents [2,3]. Therefore, researching the fault diagnosis technology for the failure of the main pump motor shielding sleeve helps to improve the operational reliability of the main pump motor.

As a complex mechanical device, the main pump motor often suffers from the following faults: air gap eccentricity, stator faults [4], bearing faults [5,6,7], rotor faults [8,9], and shielding sleeve faults. At present, the diagnostic methods for air gap eccentricity, stator faults, bearing faults, and rotor faults are relatively mature, but there is a lack of diagnostic methods for the failure of the shielding sleeve. Research on the shielding sleeve mainly focuses on the design level [10]. Machine learning algorithms are widely used in the field of main pump motor fault diagnosis. He et al. obtained vibration data of the canned motor pump in fault states through simulation tests, then extracted the characteristic quantities of the vibration data, and finally completed the fault diagnosis of the canned motor pump using the Random Forest algorithm [11]. Sunal et al. compared different fault diagnosis methods for canned motor pumps based on machine learning and analyzed the advantages of using the motor current signature analysis method for fault diagnosis as well as the limitations in obtaining fault diagnosis data [12]. Ma et al. used the time–frequency domain features of the acceleration sensor as characteristic parameters and completed the online fault diagnosis of the centrifugal pump in the nuclear power plant using the wavelet packet decomposition and Random Forest methods [13]. However, since machine learning requires manual feature selection, it limits the accuracy of feature extraction and makes it difficult to handle fault situations with high uncertainty [4].

Convolutional neural networks have the ability to learn autonomously and perform parallel processing, and they show unique advantages in dealing with non-linear and high-uncertainty problems. Therefore, they have been widely used in the field of fault diagnosis [14,15]. Traditional convolutional neural networks often use single-scale convolution kernels. However, compared to single-scale convolution kernels, multi-scale convolution kernels have stronger adaptive feature extraction capabilities, resulting in better generalization and accuracy [16,17]. Wang et al. proposed a convolutional neural network with feature alignment, which achieved multi-scale feature extraction of vibration signals, thus realizing the fault diagnosis of turbines [16]. Chen et al. proposed a multi-scale neural network with feature alignment, which achieved the fault diagnosis of bearings under different working conditions [17]. With the development of deep learning methods, multi-source data fusion methods have emerged to solve the problems of poor robustness of single data sources, the inability to distinguish similar fault modes, and sensitivity to noise and interference. The main fusion methods include data fusion [18,19] and feature fusion [20,21,22]. Xie et al. applied a multi-signal-to-RGB-image conversion method based on principal component analysis to fuse multi-signal data into three-channel red–green–blue (RGB) images. Then, they used a CNN with a residual network to achieve the fault diagnosis of mechanical equipment [18]. Ma et al. fused various sensor data, such as vibration, temperature, and current, and used the MSK-CNN model to automatically extract and analyze features, which improved the accuracy and robustness of synchronous motor fault diagnosis [19]. Fan et al. proposed a multi-scale feature fusion method based on a residual network. By fusing feature information at different scales and leveraging the deep learning ability of the residual network, they improved the accuracy of fault feature extraction and the reliability of fault identification [20]. The above-mentioned multi-source data fusion methods often only consider either data fusion or feature fusion without comprehensively considering the multi-scale features of data and their collaborative effects in the fusion process.

The internal structure of the shielding sleeve is compact, making it difficult to install additional sensors to obtain the characteristic data of the shielding sleeve. Therefore, only the performance curves (voltage, current, torque, and speed) of the main pump motor can be used as the diagnostic input data. The failure of the shielding sleeve has an insignificant impact on a single performance curve. Deeper multi-scale feature extraction of data and multi-source data fusion are required to more comprehensively reflect the failure of the shielding sleeve. Therefore, this paper proposes a multi-source data fusion fault diagnosis method for the main pump motor shielding sleeve under limited measurements. First, a multi-scale convolutional neural network based on the attention mechanism (AM-MSCNN) model is constructed. The multi-scale features of the input signal are extracted through convolution kernels of different scales, and the spatial attention mechanism and channel attention mechanism are used to weight and connect the importance of features at each scale, enhancing the feature extraction and fusion capabilities of the model. Then, on the basis of the AM-MSCNN, a convolutional neural network structure based on the attention mechanism and multi-scale multi-source data fusion (AM-MSMDF-CNN) is designed to achieve multi-source fusion of the data from current, voltage, torque, and speed sensors. Finally, the proposed method was verified using the datasets of shield sleeve failures from finite element simulations and small-scale prototype tests and was compared to other common fault diagnosis methods.

## 2. Multi-Scale Convolutional Neural Network Based on Attention Mechanism

### 2.1. Attention Mechanism Module

**Figure 1:**
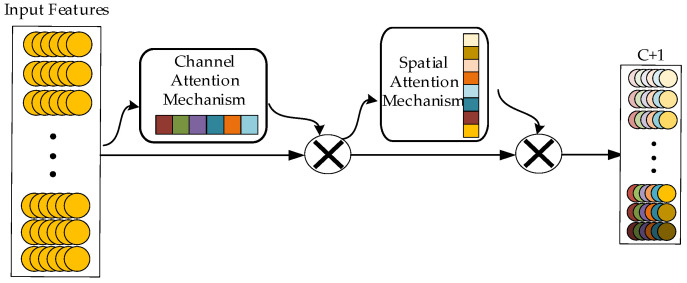
Structure diagram of the attention module.

In convolutional operations, feature maps generated by different convolutional kernels contribute differently to the final classification results. Therefore, the attention mechanism aims to enhance the network’s sensitivity to features generated by different convolutional kernels, adaptively distinguishing their importance. The structure of the attention module is shown in Figure 1. It consists of a channel attention module and a spatial attention module connected in series, combining the advantages of both channel and spatial attention while considering the interaction of information in both channel and spatial dimensions. This makes it superior to using either a single-channel attention module or a spatial attention module alone.

**Figure 2:**
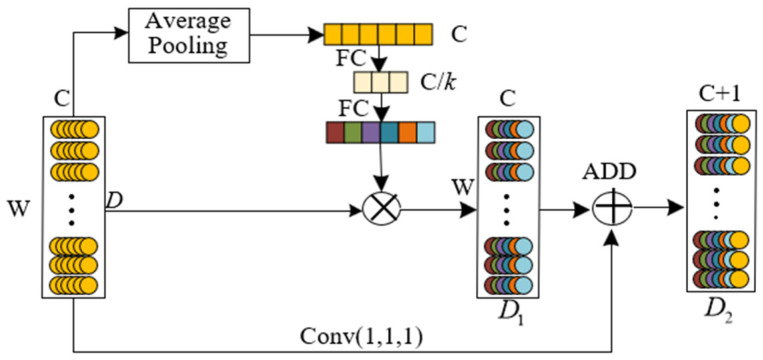
Scale attention mechanism module.

Figure 2 shows the channel attention module. First, assume that the input is *D* = [*d*_1_, *d*_2_, …, *d_C_*], which consists of *C* feature maps. These feature maps are generated by *C* convolution kernels, and the length of each feature map is *W*. The feature maps are compressed into a vector, *Z*, corresponding to the convolution kernels, *C*, through average pooling. The *i* element of *Z* can be calculated as:

(1) $$z_{i}=Avgpool(d_{i})=\frac{1}{W}\sum_{j=1}^{W}d_{i}(j)$$

where *W* denotes the feature length. This average pooling operation compresses global information into a single channel, with *z_i_* representing the channel-wise condensed information after average pooling.

Subsequently, the averaged information is processed through two fully connected layers to generate the corresponding evaluation vector. The weighted features are obtained by performing element-wise multiplication between this evaluation vector and the original features *D*, formulated as:

(2) $$z^{\prime }=\mathrm{FC}(z,C/k),z^{\prime \prime }=\mathrm{FC}(z^{\prime },C)$$

(3) $$D_{1}=D\cdot z^{\prime \prime }=[d_{1}z_{1}^{\prime \prime },d_{2}z_{2}^{\prime \prime },\cdots ,d_{C}z_{C}^{\prime \prime }]$$

where *z*′ represents the feature after dimensionality reduction and *z*″ is an evaluation vector of feature *D*. *D*_1_ denotes the feature output by the channel attention mechanism.

Since feature calibration will lead to a decrease in the response value of deep features, a 1 × 1 one-dimensional convolution is used to process the original features to improve the optimization feasibility and retain the original information. The final output is as follows:

(4) $$D_{2}=conc(D_{1},\mathrm{Conv}(D))$$

**Figure 3:**
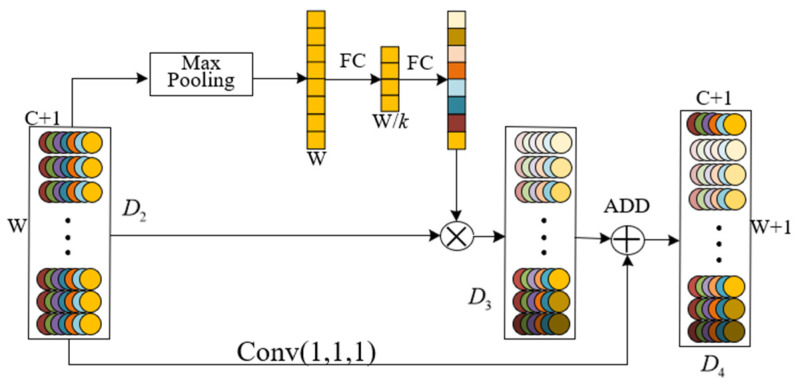
Spatial attention mechanism module.

Figure 3 shows the spatial attention module. Its principle is essentially the same as that of the channel attention mechanism. The former applies attention mechanism weighting on the width *C* of the feature map, while the spatial attention mechanism applies attention mechanism weighting on the length *W* of the feature map.

### 2.2. Multi-Scale Convolutional Neural Network

Convolutional Neural Networks (CNNs) simulate the structure and function of biological visual systems to perform feature extraction and learn deep learning models, and they are widely used in image recognition and processing. The basic components of CNN include a convolutional layer, a pooling layer, and a fully connected layer, as described in detail below.

The convolutional layer primarily performs convolution operations on the input data to extract a series of feature maps. It is typically composed of a set of learnable kernels and biases. The input into a neuron in the convolutional layer can be obtained by computing the convolution between the weights and the receptive field, which can be expressed as follows:

(5) $$y_{k,j}^{c}=b_{j}^{k}+\sum_{i=1}^{N}w_{i,j}^{k}*y_{k-1,i}^{p}$$

where $y_{k,j}^{c}$ represents the convolution result of the *j* channel in the *k* convolutional layer, $w_{i,j}^{k}$ denotes the kernel of the *k* convolutional layer, $b_{j}^{k}$ signifies the bias of the *j* channel in the *k* convolutional layer, ∗ denotes the convolution operation, and $y_{k-1,i}^{p}$ represents the convolution result of the *i* channel in the (*k* − 1) layer.

After the convolution operation is completed, the activation function determines whether to activate the neurons in the convolutional layer. The activation function helps alleviate the vanishing gradient problem and accelerates convergence. The commonly used activation function is ReLU; so, the output of the convolutional layer l can be expressed as:

(6) $$y_{l,j}^{o}=\max[0,y_{l,j}^{c}]=\left\{\begin{array}{l} y_{l,j}^{c},y_{l,j}^{c}\geq 0\\ 0,y_{l,j}^{c}<0 \end{array}\right.$$

where $y_{l,j}^{o}$ represents the output of the *j* channel in the *l* convolutional layer and max[·] is the activation function.

The pooling layer is typically applied after the convolutional layer to further extract features. Common types of pooling include average pooling, max pooling, and logarithmic pooling. Max pooling is better at extracting key features and is often used in classification tasks as it enables faster convergence. Therefore, this paper adopts max pooling, and its expression is as follows:

(7) $$y_{l,j}^{p}=\max[w(a_{1},a_{2})\cap y_{l-1,j}^{o}]$$

where $w(a_{1},a_{2})$ represents the pooling window, *a*_1_ and *a*_2_ correspond to the dimensions of the pooling window, ∩ indicates the overlap between the pooling window and the channel output, and $y_{l-1,j}^{o}$ denotes the output of the *j* channel in the (*l* − 1) layer.

**Figure 4:**
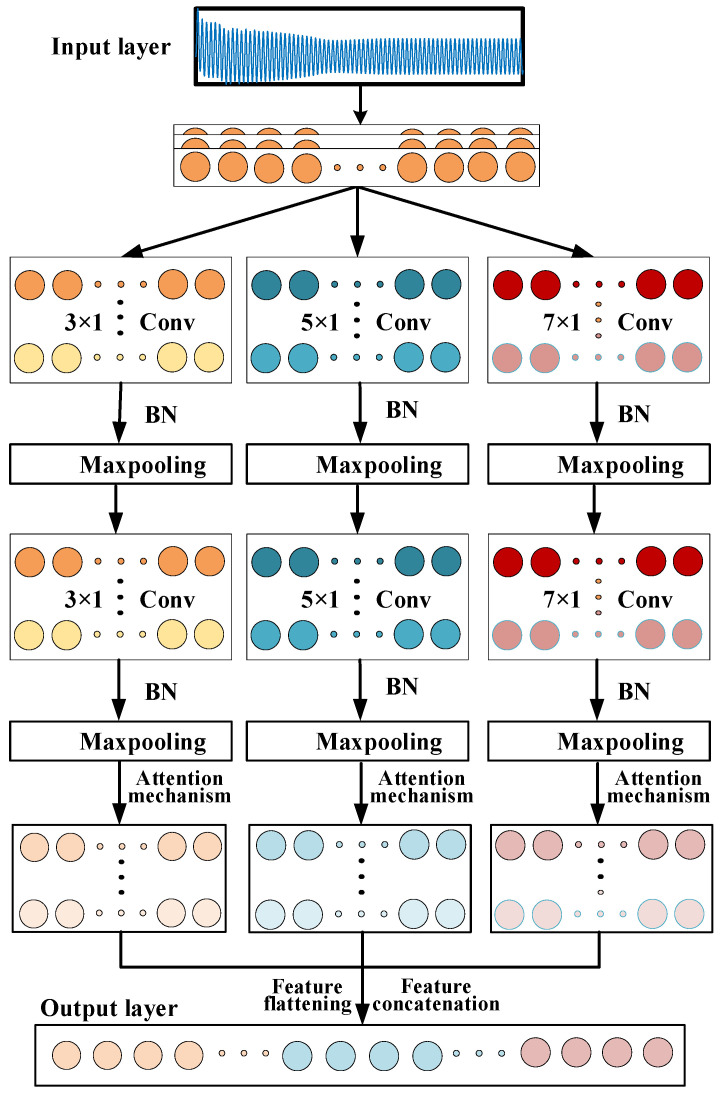
Structure diagram of multi-scale convolutional neural network based on attention mechanism.

When extracting feature information from input signals, a multi-scale convolutional neural network can capture features of input data at different scales, thereby maximizing the acquisition of more detailed signal characteristics. In the main pump motor, simulations have shown that the failure of the shielding sleeve has a minor impact on motor performance. Therefore, a multi-scale convolutional neural network is adopted to capture and extract feature information from input data at different scales to the greatest extent, which can improve the diagnostic accuracy and convergence speed of the model. However, multi-scale convolutional neural networks have certain limitations in terms of information focus and computational efficiency. Hence, this paper proposes a multi-scale convolutional neural network based on an attention mechanism. As shown in Figure 4, it includes an input layer, a multi-scale feature extraction layer, an attention mechanism, and an output layer. The input layer receives one-dimensional time-series signals as input; the multi-scale feature extraction layer includes convolutional kernels of three different scales, namely, 3 × 1, 5 × 1 and 7 × 1, as well as batch normalization (BN) and max pooling layers. The original one-dimensional time-series signals are first transformed into multiple feature maps through convolutional layers of different scales; BN is applied to normalize the data, preventing gradient vanishing issues; and max pooling is used to reduce the dimensionality of the feature maps while retaining the most important feature information. The attention layer dynamically enhances the weights of key feature maps, thereby better focusing on important features when fusing multi-scale features. The output layer flattens the attention-weighted feature data into a one-dimensional array through a flatten layer and performs feature concatenation for subsequent processing.

## 3. Convolutional Neural Network Based on Attention Mechanism and Multi-Scale Multi-Source Data Fusion

**Figure 5:**
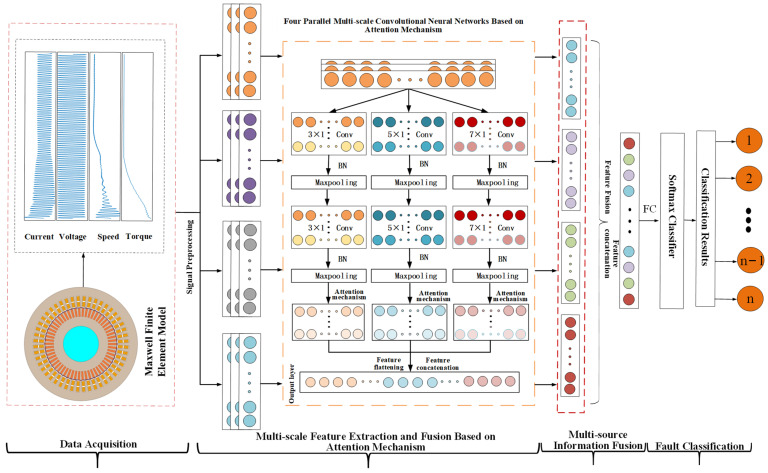
Flowchart of fault diagnosis based on attention mechanism and multi-scale multi-source data fusion.

As illustrated in Figure 5, this paper proposes the AM-MSMDF-CNN fault diagnosis process. Initially, a finite element model of the main pump motor shield failure is established using Ansys 2022 R1/Maxwell, and a series of datasets for the main pump motor shield failure are simulated by setting different operating conditions, various failure modes, and different degrees of failure. Subsequently, the preprocessed data are input into the AM-MSCNN for multi-scale feature extraction, batch normalization, max pooling, attention mechanism weighting, and multi-scale multi-feature fusion. The concatenation function is utilized to complete the feature stitching, further fusing the characteristics of torque, speed, voltage, and current. Finally, the BP algorithm and cross-entropy loss function are employed to diagnose and classify the fused features, thereby accomplishing the fault diagnosis.

### 3.1. Input Data Selection

The input signals have a significant impact on motor fault diagnosis, and research in this field extensively relies on electrical and mechanical signals. The sources of datasets can be divided into two categories: actual/experimental machines [23,24,25,26] and machine simulation software generation [27,28]. Actual/experimental machines can provide real and accurate fault signals, but they require substantial cost and time investment; machine simulation software offers the advantages of low cost and high flexibility, but the data are less realistic and precise. In addition to building datasets independently, some researchers also opt to use open-source datasets [29,30]. Since it is challenging to simulate various failure scenarios of the main pump motor shield sleeve in real-world conditions and the cost of simulation is too high, it is also difficult to collect data under different operating conditions of failures. To address this issue, the advantages and disadvantages of simulated and actual generated datasets were comprehensively compared [28], as shown in Table 1, the symbols “√” and “×” in Table 1 represent meeting and not meeting the corresponding conditions respectively. By comprehensively considering the characteristics of the actual and simulated datasets, this paper selects the simulated dataset of a large-scale motor and the experimental data of a small-scale equivalent motor as the diagnostic objects.

Additionally, the selection of input signals was based on the following characteristics: a. the ease of data acquisition; b. the relevance to shield sleeve failure; c. the advantages of multi-source heterogeneous data; and d. consistency with other studies.

Based on the above four points, the input signals for shield sleeve failure were determined to be current, voltage, torque, and speed. Current and voltage can be collected using conventional electrical measurement equipment, while torque and speed can be monitored through sensors or existing control systems. Shield sleeve failure may cause changes in the electrical and mechanical performance of the motor, thereby affecting parameters such as current, voltage, torque, and speed. In the existing literature, parameters such as current, voltage, torque, and speed are commonly used in motor fault diagnosis research, providing a certain reference value and comparability.

### 3.2. Modeling of the Main Pump Motor Considering the Failure of the Shielding Sleeve

In order to obtain the simulated dataset after the failure of the main pump motor shielding sleeve, a finite element model of the main pump motor considering the shielding sleeve failure was established. Based on the actual rated parameters of the main pump motor, other parameters of the main pump motor were designed, as shown in Table 2.

**Figure 6:**
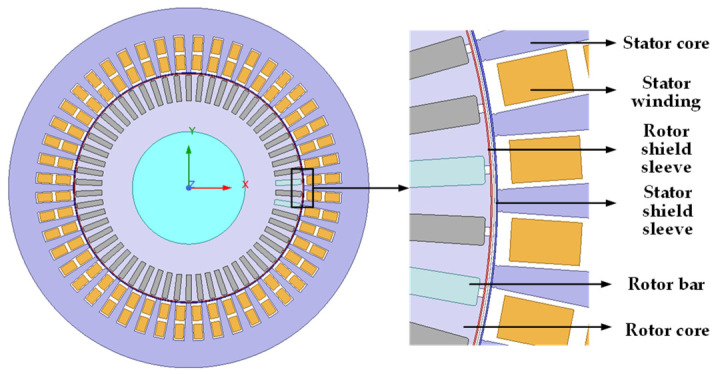
Finite element model of the main pump motor.

After determining the parameters of the main pump motor, a finite element model of the main pump motor was established using Ansys 2022 R1/Maxwell software, as shown in Figure 6. The motor used in this paper is a 785 kW/130 kW squirrel-cage shielded induction motor, and the speed control method employed is a 4/8 pole changing speed control.

**Figure 7:**
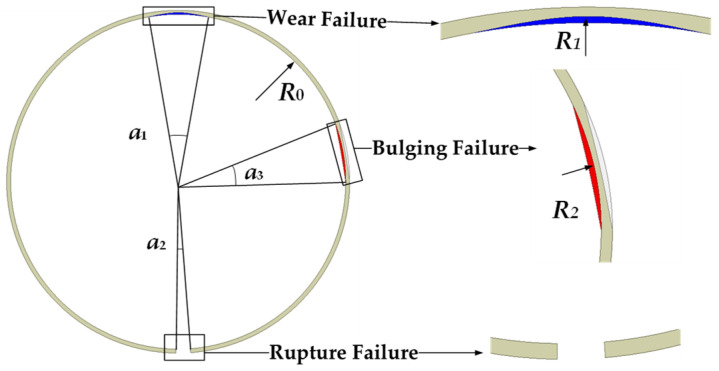
Shielding sleeve failure model.

In order to better simulate different fault states of the main pump motor shield can, a parametric method is used to model the motor and the shield can; for ease of control, scripts are employed to manage the variation of variables, thereby collecting the required dataset. Figure 7 shows the parametric model of the shielding sleeve failure, where *a*_1_, *a*_2_, and *a*_3_ respectively represent the circumferential angle ranges of wear, bulging, and rupture of the shield can and Δ*R*_1_ = *R*_1_ − *R*_0_ and Δ*R*_2_ = *R*_2_ − *R*_0_ respectively represent the changes in the maximum displacement points compared to the normal state during wear and bulging.

The boundary condition of the motor’s finite element model uses a vector boundary. The vector magnetic potential at the outer diameter of the motor stator is set to 0. The excitation source constraint adopts a voltage constraint. The motion constraint takes into account the transient process of the motor’s motion to simulate the motor’s starting process.

### 3.3. Shielding Sleeve Failure Data Collection and Preprocessing

In the previous section, a main pump motor model considering the failure of the shielding sleeve was established. This model was used to simulate normal operating conditions and three different fault conditions of the shielding sleeve (bulging, wear, and rupture). Additionally, simulations were conducted under two different operating conditions and two different levels of fault severity, where smaller numbers represent lighter fault conditions and larger numbers represent more severe fault conditions, thus dividing the failure dataset into 14 distinct classes, as shown in Table 3. The Maxwell model was able to collect four types of simulation data (current, voltage, torque, and speed), generating eight output signals for the aforementioned 14 classes. For each of these data types, the motor’s operation under a 4-pole condition was simulated for the first 1.5 s with a time step of 0.75 milliseconds and under an 8-pole condition for the first 2 s with a time step of 1 millisecond. Therefore, each sample is represented by one-dimensional data, which consists of 2001 × 8 points and all data were saved into CSV files. Each sample was then individually separated from the CSV files and labeled, as shown in Table 3. Each time-domain signal (*V*_a_, *V*_b_, *V*_c_, *I*_a_, *I*_b_, *I*_c_, *T*, and *S*_p_) was normalized by dividing by their rated values and finally used as input for the CNN for training and testing purposes.

**Figure 8:**
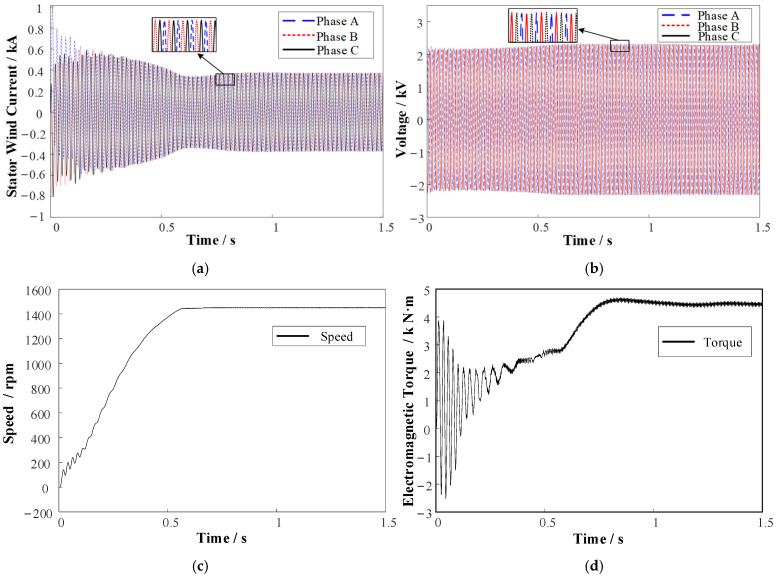
Data samples of induction motor under normal conditions. (**a**) Three-phase stator winding currents. (**b**) Three-phase voltages. (**c**) Rotational speed. (**d**) Electromagnetic torque.

Considering the inconsistency in different failure modes of the shielding sleeve, the failure variables of the parameterized shielding sleeve model from the previous section were controlled. In the case of bulging failure, it is necessary to ensure that the bulge does not come into contact with the rotor to avoid collisions. Therefore, the range of the bulge thickness Δ*R*_2_ was set to 0.1–1.4 mm as the air gap thickness was 1.5 mm, preventing the bulge thickness from exceeding the length of the air gap. For wear failure, the thickness of the shielding sleeve must be considered; so, the range of wear thickness Δ*R*_1_ was set to 0.1–0.4 mm given that the shielding sleeve thickness was 0.5 mm. The ranges for *a*_1_, *a*_2_, and *a*_3_ were set to 1–50°. Each type of data was simulated using a wide range of values, resulting in a relatively extensive dataset. As shown in Figure 8, a sample of the dataset was obtained through Maxwell simulation.

### 3.4. Multi-Source Data Fusion Method

The multi-source data fusion process is divided into two stages, and an attention mechanism module is also introduced during the fusion process. In the first stage, after multi-scale feature extraction based on the attention mechanism, features of different scales are first flattened into one-dimensional vectors and then fused together through feature concatenation. The second stage of fusion involves the feature fusion of four channels (current, voltage, speed, and torque). In this process, the conc function is used to directly concatenate features from different data sources, thereby achieving the fusion of data from different channels, as shown in Equation (8).

(8) $$F=Conc(F_{1},F_{2},F_{3},F_{4})$$

where *F_i_* represents the feature extraction results of the multi-scale convolutional neural network based on the attention mechanism for different channels.

### 3.5. Fault Classification

Similar to traditional fully connected networks, the AM-MSMDF-CNN modifies the parameters of each layer through the BP algorithm and a strategy of minimizing cross-entropy loss. Additionally, the Adam optimizer, which features adaptive learning rates and bias correction, is employed to facilitate parameter updates during the training process. This approach enables rapid convergence of the training process, as illustrated in Equations (9)–(13):

(9) $$L=-\frac{1}{n}\sum_{i}^{n}\sum_{j=1}^{k}p_{i,j}\log({\hat{q}}_{i,j})$$

(10) $$m_{t}=\beta_{1}m_{t-1}+(1-\beta_{1})(\nabla L(\theta_{t}))$$

(11) $$v_{t}=\beta_{2}v_{t-1}+(1-\beta_{2}){\left(\nabla L(\theta_{t})\right)}^{2}$$

(12) $$m_{t}^{\prime }=\frac{m_{t}}{1-\beta_{1}^{t}},v_{t}^{\prime }=\frac{v_{t}}{1-\beta_{2}^{t}}$$

(13) $$\theta_{t+1}=\theta_{t}-\eta \frac{m_{t}^{\prime }}{\sqrt{v_{t}^{\prime }}+\varepsilon }$$

where *L* represents the loss value; *n* is the size of the input sample; *p* is the true label; *q* is the actual classification result output by Softmax; *t* is the number of training iterations; *m_t_* and *v_t_* denote the first and second moment estimates, respectively; *m_t_*′ and *v_t_*′ represent the bias-corrected estimates; *ε* is a very small constant to prevent division by zero error; *θ_t_* represents the model’s training parameters; and *β*_1_ and *β*_2_ are the decay rates for the momentum and the squared gradient, respectively.

## 4. Experimental Results and Analysis

### 4.1. Experimental Verification and Analysis Based on Finite Element Modeling Data

#### 4.1.1. Performance Testing of AM-MSMDF-CNN Based on Modeling Data

To validate the effectiveness of the proposed AM-MSMDF-CNN model, the simulation data collected in the previous section were utilized, with the data divided into an 80% training set and a 20% test set for model evaluation. The model was tested and compared to traditional CNN methods. The experiment employed accuracy, loss curves, and confusion matrices as evaluation metrics to comprehensively assess the classification performance of the model.

**Figure 9:**
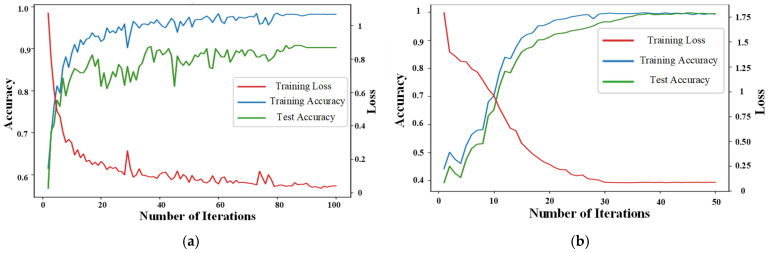
Loss and accuracy curves. (**a**) 1D-CNN. (**b**) AM-MSMDF-CNN.

The loss and accuracy curves of the 1D-CNN model during training and testing are presented in Figure 9a. The 1D-CNN model exhibits several notable shortcomings in its training performance. First, the model’s convergence speed is slow, indicating that more iterations are required to achieve satisfactory performance, which results in longer training times. Second, the accuracy shows certain fluctuations throughout the training process, which is due to the model’s insufficient extraction of data features, failing to stably capture and utilize the hidden characteristics in the data. In contrast, the AM-MSMDF-CNN model demonstrates significant advantages in its training curves. As illustrated in Figure 9b, the loss of the AM-MSMDF-CNN decreases rapidly, while the accuracy steadily increases, and its convergence speed is much faster than that of the 1D-CNN. Due to its structure based on the attention mechanism for multi-scale feature extraction and multi-source fusion, not only is the convergence speed accelerated and fluctuations reduced but the training time is also significantly shortened while ensuring model accuracy. Specifically, the AM-MSMDF-CNN can quickly identify key features in the data at the early stages of training, thereby reducing unnecessary training time and improving efficiency. Its multi-level feature fusion strategy ensures high efficiency and stability when dealing with complex data, enabling it to effectively handle multi-scale features and multi-source data.

**Figure 10:**
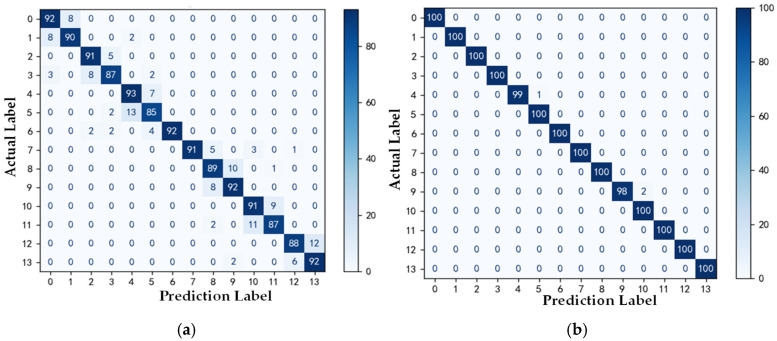
Confusion matrix of the test set. (**a**) 1D-CNN. (**b**) AM-MSMDF-CNN.

As shown in Figure 10a,b, the red curve represents the loss value, corresponding to the vertical axis on the right side, and the green and blue curves represent the accuracy rates, corresponding to the vertical axis on the left side. The confusion matrix results of the 1D-CNN and AM-MSMDF-CNN are presented in Figure 10a and Figure 10b respectively. From the figure, it can be observed that the accuracy of the 1D-CNN is only 90%, while the accuracy of the AM-MSMDF-CNN reaches 99.8%, which is 9.8% higher than that of the 1D-CNN.

#### 4.1.2. Comparison of Data Fusion Effects for Different Types of Data

In order to compare the fusion of various types of information during the information fusion process and the impact of different information on the overall model, we analyzed five different combination scenarios, as shown in Table 4.

**Figure 11:**
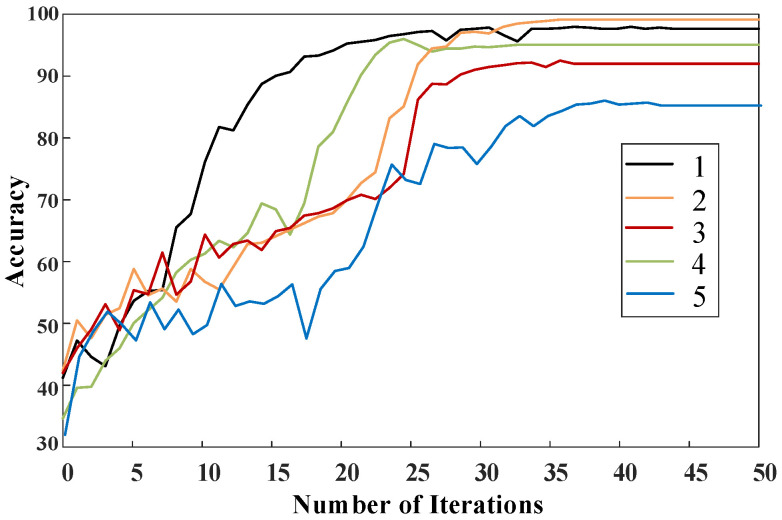
Diagnostic accuracy of different information fusion combinations based on prototype data.

As can be seen from Table 4, different signal fusion combinations exhibit varying levels of accuracy, allowing for the determination of importance ranking in diagnosing faults in the shielding sleeve. To more intuitively observe the changes in accuracy during the training process with different data fusions, a comparison of fault diagnosis accuracy is illustrated in Figure 11. From both the table and the figure, it is evident that when torque is included, the accuracy is above 90%, whereas without torque, it is only 85%. In the absence of current, speed, and voltage, the accuracies are 92%, 95%, and 99.1%, respectively. From the correlation coefficients and *p*-values in Table 5, it can be seen that the order of the four signals in terms of their correlation with the diagnosis, from high to low, are torque, current, rotational speed, and voltage, which is consistent with the judgment result based on the accuracy. Therefore, it can be inferred that the approximate order of importance for diagnosis is torque, current, speed, and voltage. Additionally, these signals possess complementary characteristics, and their fusion can enhance the accuracy of shielding sleeve fault diagnosis.

#### 4.1.3. Comparison of Different Algorithm Performances

To further validate the fault diagnosis efficacy of the proposed model, the AM-MSMDF-CNN was compared to other networks. For this study, the 1D-CNN, Bagging, Random Forest, and SVM were selected as comparative models for experimentation. All four models utilized the same dataset as the AM-MSMDF-CNN for their experiments.

In the experiments, identical data preprocessing steps and feature engineering methods were applied across all models to facilitate a direct comparison of their performances. The hyperparameters of the models were optimized through grid search and cross-validation to achieve the best possible experimental outcomes.

As shown in Table 6, the accuracy rates of the 1D-CNN, Bagging Ensemble Learning, Random Forest, and SVM are 91%, 93.4%, 95.2%, and 90.2%, respectively; these are significantly lower than that of the AM-MSMDF-CNN. This is attributed to the fact that the AM-MSMDF-CNN is equipped with multi-scale convolutional layers and a hierarchical information fusion strategy, enabling it to extract and comprehend the complex features of input signals more comprehensively. In fault diagnosis, the AM-MSMDF-CNN outperforms other models in both accuracy and robustness. The experimental results demonstrate that under various fault modes, the average accuracy of the AM-MSMDF-CNN is approximately 5–10% higher than that of the other models.

### 4.2. Experimental Verification and Analysis Based on Small-Scale Prototype Test Data

#### 4.2.1. Small Prototype Test Platform and Relevant Parameters

**Figure 12:**
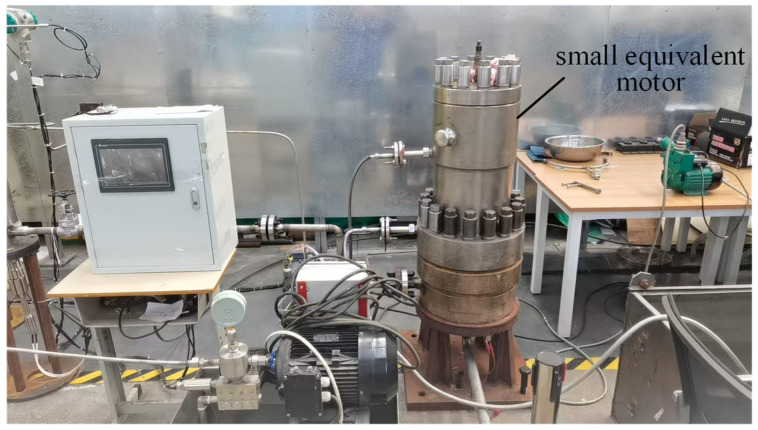
Test platform for the small prototype.

This experiment uses the main pump motor as a prototype to design a small equivalent canned motor. The equivalent circuit, shielding sleeve material, shielding sleeve thickness, and speed-regulation method of the small motor are the same as those of the main pump motor. The test platform is shown in Figure 12, and the basic parameters of the motor are shown in Table 7.

Since the coolant may damage the prototype when the shielding sleeve ruptures, the performance tests of the shielding sleeve failure test of the prototype were only conducted under three conditions: normal, worn, and bulged. The performance curves of the motor were measured under both high-speed and low-speed operating conditions.

#### 4.2.2. Performance Test of AM-MSMDF-CNN Based on Experimental Data

The collected prototype test data were preprocessed, and the following shielding sleeve failure dataset was obtained, as shown in Table 8. Compared to the simulation dataset, the failure data of the shielding sleeve rupture were missing. Since simulating the rupture of the shielding sleeve would damage the prototype, the shielding sleeve rupture test was not carried out.

**Figure 13:**
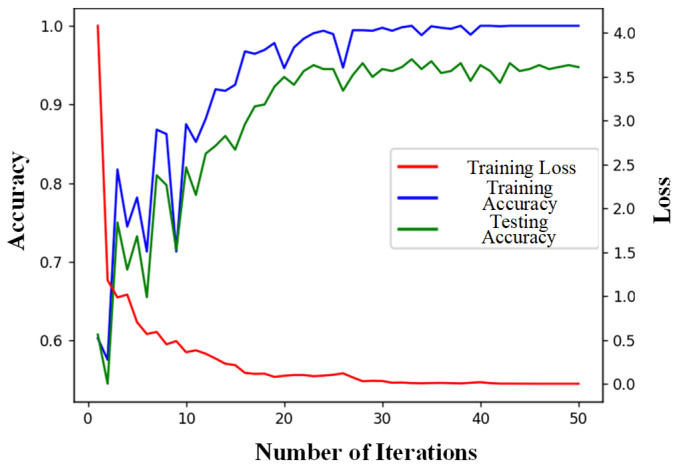
Loss value and accuracy curves.

The AM-MSMDF-CNN algorithm was used to test the prototype test dataset. As shown in Figure 13, the accuracy of the AM-MSMDF-CNN for the training set can still reach 100%, but the accuracy of the test set is only 95.1%, which is 5% lower than that of the simulation dataset.

#### 4.2.3. Comparison of Data Fusion Effects and Algorithms for Different Types of Data

Using a method similar to that for analyzing the simulation dataset, the data fusion effects of different types of data were analyzed, with the results shown in Table 9 and Table 10.

It can be seen from the diagnostic accuracy under different signal fusions in Table 9 and the relationship between different signals and diagnostic accuracy in Table 10 that the signals are ranked in descending order of importance for diagnosis as torque, current, rotational speed, and voltage.

It can be seen from Table 11 that the diagnostic accuracy of different algorithms for the experimental data of the prototype platform is generally lower than that for the simulation dataset. This may be because in the actual platform, the motor is affected by some other external factors, such as voltage fluctuations and temperature, which generate noise and reduce the diagnostic accuracy. However, by comparing the accuracy of different algorithms, it was found that the accuracy of other algorithms drops significantly, while the accuracy of the AM-MSMDF-CNN drops less, indicating that it has a certain anti-interference ability.

## 5. Conclusions

This paper proposes a fault diagnosis method based on a multi-scale and multi-source data fusion convolutional neural network to complete the fault diagnosis of the shielding sleeve failure of the main pump motor. This method takes the torque, rotational speed, current, and back electromotive force of the main pump motor as input signals, successfully extracts multi-scale features, uses the attention mechanism to fuse these features, and then completes the fault diagnosis of the shielding sleeve. The following conclusions are drawn:

(1) The AM-MSCNN utilized in this study effectively extracts multi-scale features from the dataset. Its attention mechanism adeptly addresses the issue of information loss during the fusion of signals at different scales and resolves the feature weighting problem, thereby accentuating significant features and diminishing redundant ones.
(2) The experiments reveal that different signals exhibit varying degrees of discernibility for the failure of the motor shield sleeve in the main pump. There are complementary characteristics among different signals, and the fusion of multi-source data contributes to enhancing the accuracy and robustness of fault diagnosis.
(3) By comparing the diagnostic accuracies of different algorithms, it was found that for both the simulation data and experimental data, the accuracy of the AM-MSMDF-CNN is 5–10% and 10–15% higher than that of other models, respectively.

## Figures and Tables

**Table 1:** Comparison of real dataset and simulated dataset.

	Experimental Data	Simulated Data
Machine Modeling Conditions	× Difficult to adapt to different machines and fault types	√ Capable of adapting to different machines and various types of faults × Requires complex modeling to simulate real-world conditions
Operating Conditions	× Difficult to achieve various operating conditions	× Requires complex modeling to simulate real-world conditions
Dataset	× Under fault conditions, a long runtime is required to obtain a large dataset during stable operation × Fault simulation on large machines is too costly, making it difficult to obtain a large dataset	Under fault conditions, it is easy to obtain a large dataset without causing damage to the machine √ Can simulate the operation of large machines without the need for actual testing
Fault Detection Capability	√ Providing actual measured data for training can enhance the accuracy of fault detection on real machines	× Using simulated data for training, which does not fully equate to real data, it poses challenges to the fault detection work of actual machines.

**Table 2:** Main parameters of the main pump motor.

Parameters	Value	Parameters	Value
Rated Power/kW	785/130	Rated Torque/N	5100/1650
Rated Voltage/V	3000	Rated Current/A	270/47
Pole Pairs	4/8	Shielding Sleeve Thickness/mm	0.5
Rated Speed/rpm	1450/725	Shielding Sleeve Material	Hastelloy C-276
Stator Inner (Outer) Diameter/mm	410 (640)	Number of Rotor Slots	58
Number of Stator Slots	48	Iron Stator Core Length/mm	1300

**Table 3:** Sample data labels.

Sample Quantity/Length	Operating Condition	Types of Shield Failures	Label
1000/2000	Rapid Startup	Bulging	0, 1
1000/2000	Rapid Startup	Wear	2, 3
1000/2000	Rapid Startup	Rupture	4, 5
500/2000	Rapid Startup	Normal	6
500/2000	Slow Startup	Normal	7
1000/2000	Slow Startup	Bulging	8, 9
1000/2000	Slow Startup	Wear	10, 11
1000/2000	Slow Startup	Rupture	12, 13

**Table 4:** Diagnostic accuracy of different information fusion combinations based on simulation data.

Serial Number	Signal Combination	Accuracy Rate/%
1	Torque, Speed, Current, and Voltage	99.8
2	Speed, Torque, and Current	99.1
3	Voltage, Speed, and Torque	92
4	Voltage, Torque, and Current	95
5	Speed, Current, and Voltage	85

**Table 5:** Correlation between different signals and the diagnostic accuracy of simulation data.

Signal Type	Correlation Coefficient	*p*-Value
Torque	0.812	0.008
Current	0.528	0.144
Speed	0.424	0.256
Voltage	0.241	0.532

**Table 6:** Comparison of accuracy rates for different fault diagnosis algorithms.

Different Algorithms	Accuracy/%
1D-CNN	90
Bagging Ensemble Learning	93.4
Random Forest	95.2
SVM	90.2
AM-MSMDF-CNN	99.8

**Table 7:** Basic parameters of the prototype.

Parameter	Value	Parameter	Value
Rated power/kW	12/2	Rated current/A	25/5.5
Rated voltage/V	380	Thickness of shielding sleeve/mm	0.5
Number of pole pairs	4/8	Material of shielding sleeve	Hastelloy C-276
Rated speed/rpm	1450/725	Axial length/mm	200
Number of stator slots	48	Number of rotor slots	44

**Table 8:** Prototype test dataset.

Sample Quantity/Length	Operating Condition	Types of Shield Failures	Label
400/2000	Rapid Startup	bulging	0, 1
400/2000	Rapid Startup	wear	2, 3
200/2000	Rapid Startup	normal	4
200/2000	Rapid Startup	normal	5
400/2000	Slow Startup	bulging	6, 7
400/2000	Slow Startup	wear	8, 9

**Table 9:** Diagnostic accuracy for different signal fusions.

Signal Combination	Accuracy (%)
Torque, Rotational Speed, Current, and Voltage	95.1
Rotational Speed, Torque, and Current	88.2
Voltage, Rotational Speed, and Torque	86.4
Voltage, Torque, and Current	85.5
Rotational Speed, Current, and Voltage	80.1

**Table 10:** Correlation between different signals and the diagnostic accuracy of prototype data.

Signal Type	Correlation Coefficient	*p*-Value
Torque	0.809	0.008
Current	0.516	0.155
Speed	0.434	0.243
Voltage	0.272	0.479

**Table 11:** Comparison of diagnostic accuracies of different algorithms.

Different Algorithms	Accuracy (%)
1D-CNN	84.5
Bagging Ensemble Learning	82.2
Random Forest	81.1
SVM	80.4
AM-MSMDF-CNN	95.1

## Data Availability

No data were used for the research described in the article.
